# First Report and Characterization of the *mcr-1* Positive Multidrug-Resistant *Escherichia coli* Strain Isolated from Pigs in Croatia

**DOI:** 10.3390/microorganisms11102442

**Published:** 2023-09-28

**Authors:** Gordan Kompes, Sanja Duvnjak, Irena Reil, Rene S. Hendriksen, Lauge Holm Sørensen, Maja Zdelar-Tuk, Boris Habrun, Luka Cvetnić, Antonela Bagarić, Silvio Špičić

**Affiliations:** 1 Department for Bacteriology and Parasitology, Croatian Veterinary Institute, 10000 Zagreb, Croatia; kompes@veinst.hr (G.K.); zdelar-tuk@veinst.hr (M.Z.-T.); habrun@veinst.hr (B.H.); lcvetnic@veinst.hr (L.C.); marijan@veinst.hr (A.B.); spicic@veinst.hr (S.Š.); 2 Research Group for Global Capacity Building, National Food Institute, Technical University of Denmark, Kemitorvet, 2800 Lyngby, Denmark; rshe@food.dtu.dk (R.S.H.); lahoso@food.dtu.dk (L.H.S.)

**Keywords:** antimicrobial resistance, *mcr-1*, MDR, *Escherichia coli*, pigs

## Abstract

The emergence and rapid spread of the plasmid-mediated colistin-resistant *mcr-1* gene introduced a serious threat to public health. In 2021, a multi-drug resistant, *mcr-1* positive *Escherichia coli* EC1945 strain, was isolated from pig caecal content in Croatia. Antimicrobial susceptibility testing and whole genome sequencing were performed. Bioinformatics tools were used to determine the presence of resistance genes, plasmid Inc groups, serotype, sequence type, virulence factors, and plasmid reconstruction. The isolated strain showed phenotypic and genotypic resistance to nine antimicrobial classes. It was resistant to colistin, gentamicin, ampicillin, cefepime, cefotaxime, ceftazidime, sulfamethoxazole, chloramphenicol, nalidixic acid, and ciprofloxacin. Antimicrobial resistance genes included *mcr-1, bla_TEM-1B_, bla_CTX-M-1_*, *aac(3)-IId*, *aph(3’)-Ia*, *aadA5*, *sul2*, *catA1*, *gyrA* (S83L, D87N), and *parC* (A56T, S80I). The *mcr-1* gene was located within the conjugative IncX4 plasmid. IncI1, IncFIB, and IncFII plasmids were also detected. The isolate also harbored 14 virulence genes and was classified as ST744 and O101:H10. ST744 is a member of the ST10 group which includes commensal, extraintestinal pathogenic *E. coli* isolates that play a crucial role as a reservoir of genes. Further efforts are needed to identify *mcr-1*-carrying *E. coli* isolates in Croatia, especially in food-producing animals to identify such gene reservoirs.

## 1. Introduction

The global rise of antimicrobial resistance (AMR) and the emergence and spread of multidrug-resistant (MDR) bacteria represent a huge public health threat. Against such Gram-negative pathogens, polymyxins have been regarded as last-resort antibiotics [1]. Polymyxins are a group of cationic polypeptide antibiotics that consist of five different compounds (polymyxin A-E). Currently, only polymyxin E (colistin) and polymyxin B have been put into clinical use. This group was discovered in 1947 as a metabolite produced by the bacterium *Paenibacillus polymyxa* (formerly known as *Bacillus polymyxa* var. colistinus) and was originally named ‘aerosporin’ [2,3]. Due to its neurotoxicity and nephrotoxicity, colistin was rarely used in human medicine and was initially restricted to ophthalmic and topical use [4,5,6]. However, it was widely used in veterinary medicine to treat infections caused by Enterobacteriaceae. The main indication for colistin use in veterinary medicine was the treatment of gastrointestinal tract infections caused by non-invasive *Escherichia* (*E*.) *coli* in pigs, poultry, cattle, sheep, goats, and rabbits. Typically, colistin products were administered orally, as a drench, in feed, in drinking water, or through milk replacer diets [7]. Colistin was thought to be a ‘safe’ antimicrobial because the prevalence of resistance remained very low. Colistin resistance was traditionally linked to the chromosomal-encoded mechanisms that involved two-component systems, phoPQ and pmrAB, and mutation in the mgrB regulator, resulting in increasing the positive charge on lipopolysaccharides (LPSs) and decreasing colistin binding isolates, leading to colistin resistance [8,9,10,11].

The first plasmid-mediated mobilize-colistin-resistant (*mcr*) gene, the *mcr-1* gene, was detected in 2015 in China [12]. The *mcr-1* gene confers colistin resistance due to the ability to encode a phosphoethanolamine transferase that modifies lipid A, reducing its attraction to colistin [13]. This discovery introduced a serious threat to public health associated with the fact of horizontal *mcr* gene transfer and the emergence of bacteria resistant to all available antimicrobials [10,14,15]. As a result, polymyxins are classified as Highest Priority Critically Important Antimicrobials (HPCIA) for human medicine by the World Health Organization (WHO) [16], and as Veterinary Highly Important Antimicrobial agents (VHIA) by the World Organization for Animal Health (WOAH) [17]. According to the Antimicrobial Advice Ad Hoc Expert Group (AMEG) categorization, polymyxins are classified as Category B with the indication ‘Restrict’ use in veterinary medicine within the EU [18].

Multiple studies have found that the *mcr-1* gene spreads rapidly in animals, humans, and the environment [15,19]. Until now, nine additional *mcr* genes encoding colistin resistance have been identified (*mcr-2* to *mcr-10*) [20,21,22,23,24,25,26,27,28]. By mid-2022, *mcr* genes in *E. coli* have been found on five continents, with a total of 54 countries reporting publications from 2018. The mcr-1 variant is the most common and widely distributed across all continents and hosts. The total crude prevalence of worldwide distribution of mcr in *E. coli* in non-clinical isolates was 6.52% and 1.76% in clinical isolates [29]. The *mcr-1* gene has been detected on plasmids of various incompatibility (Inc) groups, with IncX4, IncI2, and IncHI2 being the most common types [30,31]. There is evidence that this plasmid-borne gene is spreading rapidly among the pig population. [32].

In this study, we report the first detection of *mcr-1* plasmid gene in the MDR *E. coli* strain isolated from pig caecal content in Croatia. The aim of this research includes describing the resistance profile against several classes of antimicrobials, as well as the virulence potential of *E. coli* EC1945 isolate. 

## 2. Materials and Methods

### 2.1. Isolation and Identification

As part of Croatia’s surveillance program under Directive 2003/99/EC and Commission Implementing Decision 2020/1729/EU, an *E. coli* strain EC1945 was isolated from pig caecal content in 2021. The program, which began in 2017, specifically monitors ESBL-/AmpC-/carbapenemase producing *E. coli*. Over three years, 1028 non-duplicate pig caecal contents were examined (2017–370; 2019–396; 2021–262) using the EU Reference Laboratory–Antimicrobial Resistance (EURL-AR) protocol, which is available on the EURL-AR website (https://www.eurl-ar.eu/CustomerData/Files/Folders/21-protocols/530_esbl-ampc-cpeprotocol-version-caecal-v7-09-12-19.pdf) (accessed on 4 January 2021).

Bacterial identification was performed using the VITEK 2 system (bioMérieux, Marcyl’Etoile, France). The isolate was stored at −80 °C in the Tryptic Soy broth medium containing 30% glycerol for further study.

### 2.2. Antimicrobial Susceptibility Testing

Antimicrobial susceptibility testing (AST) was made by the broth microdilution method according to ISO 2776-1:2020 [33]. Briefly, the isolate was incubated overnight on blood agar supplemented with 5% sheep blood in an aerobic atmosphere. AST was carried out on the EUVSEC3 microplate (Sensititer, Trek Diagnostic Systems Ltd. East Grinstead, West Sussex, RH19 1XZ, UK). Demineralized water (API Suspension Medium, bioMérieux SA) was used for the preparation of 0.5 McFarland solution. A total of 50 µL of the initial solution was added in 11 mL of cation-adjusted Mueller–Hinton broth for an inoculum of 5 × 10^5^ CFU/mL. Microplates were incubated in the aerobic environment at 35 ± 1 °C for 18 ± 2 h. The susceptibility to 15 antibiotics, including azithromycin (AZI; 2–64 mg/L), amikacin (AMI; 4–128 mg/L), gentamicin (GEN; 0.5–16 mg/L), tigecycline (TGC; 0.25–8 mg/L), ampicillin (AMP; 1–32 mg/L), ceftazidime (TAZ; 0.25–8 mg/L), cefotaxime (FOT; 0.25–4 mg/L), meropenem (MERO; 0.03–16 mg/L), colistin (COL; 1–16 mg/L), tetracycline (TET; 2–32 mg/L), trimethoprim (TMP; 0.25–16 mg/L), sulfamethoxazole (SMX; 8–16 mg/L), chloramphenicol (CHL; 8–64 mg/L), nalidixic acid (NAL; 4–64 mg/L), and ciprofloxacin (CIP; 0.015–8 mg/L) was determined using the European Committee on Antimicrobial Susceptibility Testing (EUCAST) epidemiological cut-off values (ECOFFs), recommended by European Food Safety Authority (EFSA) [34].

Since the isolate was found to be resistant to cefotaxime and ceftazidime on the EUVSEC3 plate, it was further tested on the EUVSEC2 microplate containing: cefoxitin (FOX; 0.5–64 mg/L), cefepime (FEP; 0.06–32 mg/L), cefotaxime (FOT; 0.25–64 mg/L), ceftazidime (TAZ; 0.5–128 mg/L), ertapenem (ETP; 0.015–2 mg/L), imipenem (IMI; 0.12–16 mg/L), meropenem (MERO; 0.03–16 mg/L), cefotaxime + clavulanic acid (F/C; 0.06/4–64/4 mg/L), ceftazidime + clavulanic acid (T/C; 0.12/4–128/4 mg/L), and temocillin (TRM; 4–128 mg/L).

Reference strain *E. coli* ATCC 25922 was used for quality control.

### 2.3. Polymerase Chain Reaction (PCR)

Multiplex PCR for the detection of *mcr-1*–*mcr-5* variants was performed using the protocol recommended by EURL-AR [35].

### 2.4. Whole Genome Sequencing (WGS)

The *E. coli* EC1945 isolate genome was sequenced at EURL-AR, DTU, Lyngby, Denmark, under the scope of the EFSA confirmatory testing [36]. Genomic DNA was extracted using an Invitrogen Easy-DNA KitTM (Invitrogen, Carlsbad, CA, United States), and the DNA concentrations were determined using the Qubit dsDNA BR assay kit (Invitrogen). Genomic DNA was prepared for Illumina pair-end sequencing using the Illumina (Illumina, Inc., San Diego, CA, USA) Nextera XT^®^ Guide following the protocol revision C1. A sample of the pooled Nextera XT Libraries was loaded onto an Illumina MiSeq reagent cartridge using MiSeq Reagent Kit v3. The libraries were sequenced using an Illumina MiSeq platform (Illumina). The raw reads were de novo assembled using the assembler pipeline (version 1.4) available from the Center for Genomic Epidemiology (CGE) (https://www.genomicepidemiology.org/) (accessed on 25 April 2022).

The Whole Genome Shotgun project has been deposited at DDBJ/ENA/GenBank under the accession JAUTEE000000000.

### 2.5. Bioinformatics Analysis

Antimicrobial resistance genes, plasmid Inc groups, virulence factors, serotype, and multilocus sequence typing (MLST) were determined using ResFinder 4.1, PlasmidFinder 2.1, VirulenceFinder 2.0, SerotypeFinder 2.0, and MLST 2.0, respectively, using services from CGE database (https://www.genomicepidemiology.org/) (accessed on 28 April 2023).

RFPlasmids tool [37] was used to identify which contigs belonged to plasmids and which to chromosomal DNA. Any hits with an 80% probability or higher were considered to have a plasmid origin. These contigs were then screened further for AMR and virulence genes using ResFinder 4.1 and VirulenceFinder 2.0, and plasmid detection using PlasmidFinder 2.1 from the CGE server (https://www.genomicepidemiology.org/) (accessed on 14 August 2023). Additionally, all hits with 50–80% probability were also screened using the same tools. Contigs associated with plasmids were aligned with reference NCBI sequences using BioNumerics software ver. 8.1 (BioMerieux, Marcyl’Etoile, France) (OK642378.1, KU761327.1, and KX236309.1 for IncX4; MH847511 and KF362122.2 for IncI-1; CP066837.1 for IncFII; MN8163372 for IncFIB). Plasmid reconstruction and annotation were conducted using Proxee [38]. 

MLST profile was determined through the Achtman scheme analysis on a total of 7 housekeeping genes (*adk, fumC, gyrB, icd, mdh, recA, purA*) [39]. Alleles and sequence types were identified using BioNumerics (version 8.1, Applied Maths, Belgium). Alleles formed 7-digit numerical codes were used for comparisons based on categorical coefficient and UPGMA (Unweighted Pair Group Method with Arithmetic Mean). MLST results were compared to results in the PubMLST *E. coli* database (https://pubmlst.org/organisms/escherichia-spp) (accessed on 4 May 2023) [40].

The core genome MLST (cgMLST) method was used to compare alleles and complete linkage cluster analysis using BioNumerics software (version 8.1, Applied Maths, Belgium). The cgMLST results of our strain were compared with the results of strains present in the PubMLST *E. coli* database (https://pubmlst.org/organisms/escherichia-spp) (accessed on 4 May 2023) [40] based on categorical differences in alleles and complete linkage clustering analysis using BioNumerics (version 8.1, Applied Maths, Belgium).

## 3. Results

### 3.1. Antimicrobial Susceptibility Testing and Polymerase Chain Reaction (PCR)

The minimum inhibitory concentrations for EC1945 *E. coli* isolate are listed in Table 1. This strain exhibited MDR phenotype and was found to be resistant to colistin, gentamicin, ampicillin, cefepime, cefotaxime, ceftazidime, sulfamethoxazole, chloramphenicol, nalidixic acid, and ciprofloxacin. However, it remained susceptible to azithromycin, amikacin, tigecycline, cefoxitin, temocillin, ertapenem, meropenem, imipenem, tetracycline, and trimethoprim. Considering the EUCAST guidelines [41], this isolate was identified as an Extended Spectrum Beta-Lactamase (ESBL) producer due to its resistance to cefotaxime and ceftazidime, susceptibility to cefepime, and synergism between cefotaxime/clavulanic acid and ceftazidime/clavulanic acid.

Multiplex PCR analysis confirmed the presence of the *mcr-1* gene.

### 3.2. Whole Genome Sequencing and Bioinformatic Analysis

Based on the WGS analysis, it was determined that the *E. coli* EC1945 isolate had a length of 5,095,464 base pairs.

ResFinder 4.1 results revealed the presence of the *mcr-1.1* gene, which showed a 100% identity to *E. coli* strain SHP45 plasmid pHNSHP45 [12].

In addition to the *mcr-1* gene, ResFinder 4.1 detected various other resistance genes in the *E. coli* EC1945 isolate, including beta-lactams resistance genes (*bla_TEM-1B_* and *bla_CTX-M-1_*), aminoglycosides resistance genes (*aac(3)-IId, aph(3’)-Ia* and *aadA5*), sulphonamide resistance gene (*sul2*), and phenicols resistance gene (*catA1*). Mutations were also identified in *gyrA* (S83L, D87N) and *parC* (A56T, S80I) genes, which are associated with quinolones and fluoroquinolones resistance. Furthermore, the EC1945 strain contained the *sitABCD* gene, which confers disinfectant resistance (Table 1).

Four Inc groups were detected in EC1945 using PlasmidFinder 2.1, including IncX4 (Accesion no. CP002895), IncI1 (Accesion no. AP005147), IncFIB (Accesion no. AP001918), and IncFII (Accesion no. CR942285).

### 3.3. Virulence Factors

Virulence factors, identification percentage, and protein function identified in *E. coli* EC1945 isolate are listed in Table 2. *E. coli* EC1945 isolate harbored virulence genes *csgA* (curlin major subunit CsgA), *fimH* (type 1 fimbriae), *gad* (glutamate decarboxylase), *hlyE* (hemolysin E), *hlyF* (hemolysin F), *iroN* (enterobactin siderophore receptor protein), *iss* (increased serum survival), *sitA* (iron transport protein), *terC* (tellurium ion resistance protein), traT (outer membrane protein complement resistance), and four colicin coding genes (*cea*—colicin E1, *cia*—colicin Ia, *cib*—colicin Ib and *cma*—colicin M). 

### 3.4. Mobile Genetic Elements

RFPlasmids tool identified 16 contigs belonging to plasmids with >80% probability. Contigs determined as part of a plasmid origin were: 40, 51, 61.68, 79.84, 87, 100, 105, 106, 107, 110, 112, 114, 157, and 173. All hits screened with 50–80% probability gave negative results (Table 3).

Table 3 indicates that all AMR genes, except *gyrA* (p.S83L), *gyrA* (p.D87N), *parC* (p.A56T), and *parC* (p.S80I), were located on plasmids. However, quinolone and fluoroquinolone resistance were chromosomally mediated. Based on our findings, it can be concluded that the *mcr-1* gene was present on the IncX4 plasmid, while the *bla_CTX-M-1_* and *aadA5* genes were located on the IncI1 plasmid, as they were identified on the same contigs. Among the virulence genes, only *traT*, *sitA*, and *cea* were identified on contigs that belonged to plasmids.

**Figure 1:**
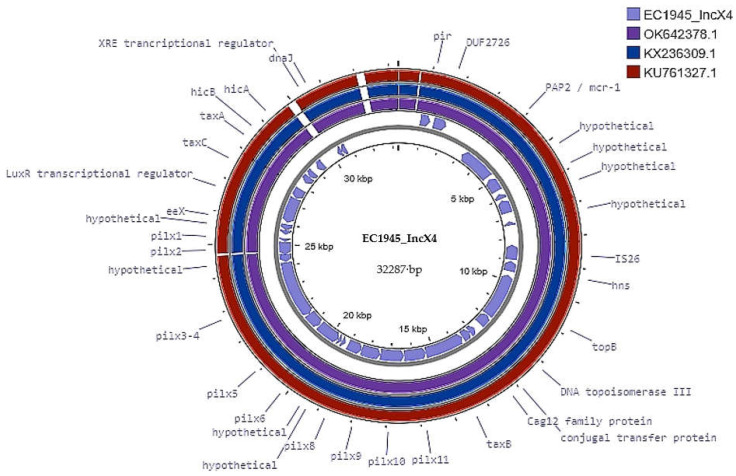
Structural comparison between *mcr-1* plasmids. The alignment includes three references (OK642378.1, KX236309.1, and KU761327.1) and IncX4 *mcr-1*-bearing plasmid found in our study (EC1945_IncX4).

Using the Proksee v1.0.0a6 tool, we were able to reconstruct and annotate the IncX4 plasmid from all the plasmid contigs, as presented in Figure 1. Unfortunately, we were unable to confidently assemble the other plasmids due to significant overlap on multiple contigs.

Through analysis of the complete plasmid sequences, it was found that the backbone of EC1945_IncX4 plasmid is highly similar to previously sequenced *mcr-1*-carrying IncX4 plasmids, with a similarity of over 98% (Figure 1).

Within the EC1945_IncX4 plasmid, we identified the *mcr-1/PAP2* cassette (2513 bp) as well as putative conjugal transfer components such as the auxiliary factor TaxA, relaxase TaxC, type IV secretion system genes (T4SS), pilX1-pilX11, and type IV coupling protein gene, taxB (T4CP). Additionally, the *ori*-T-like region contained a pair of 14-bp insert repeats (GCAGGTGAGCAAAG…CTTTGTTCACCTGA) (coordinates 27,161–27,194 bp). 

### 3.5. Multilocus Sequence Typing and Core Genome Multilocus Sequence Typing

Further analysis using MLST and serotyping revealed that the *E. coli* strain EC1945 belonged to sequence type 744 (ST744) and O101:H10 serotype (Table 4).

**Figure 2:**
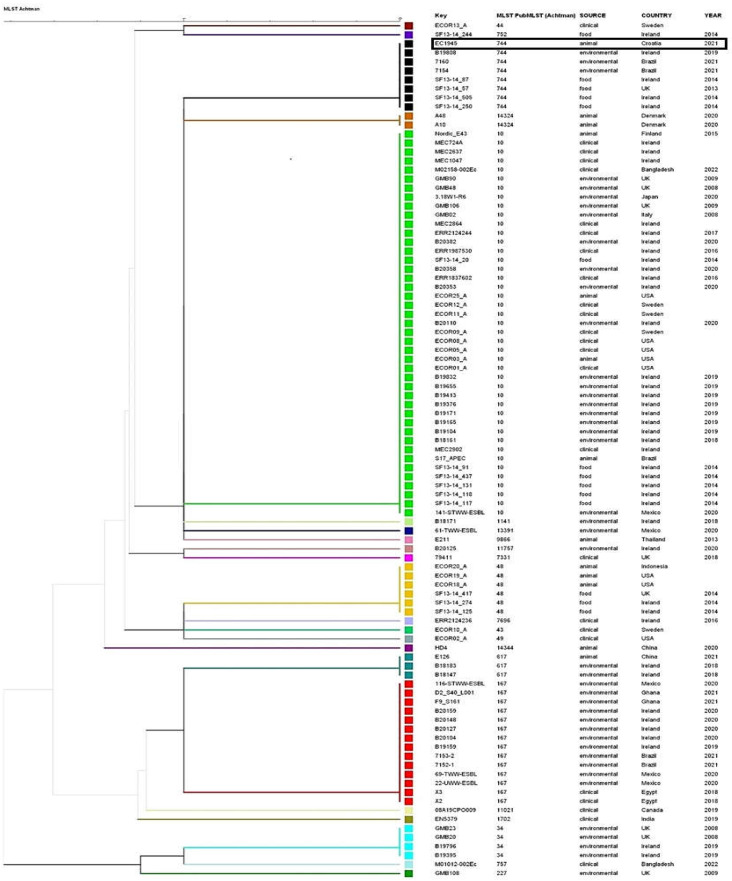
Dendrogram showing relationship of identified strain to the most related strains present in database used in this study according to MLST 7 Achtman scheme. Strains are also identified according to ST, source, country of origin species, and year of isolation. STs are designated by different colors. Isolate from this study is in rectangle.

**Figure 3:**
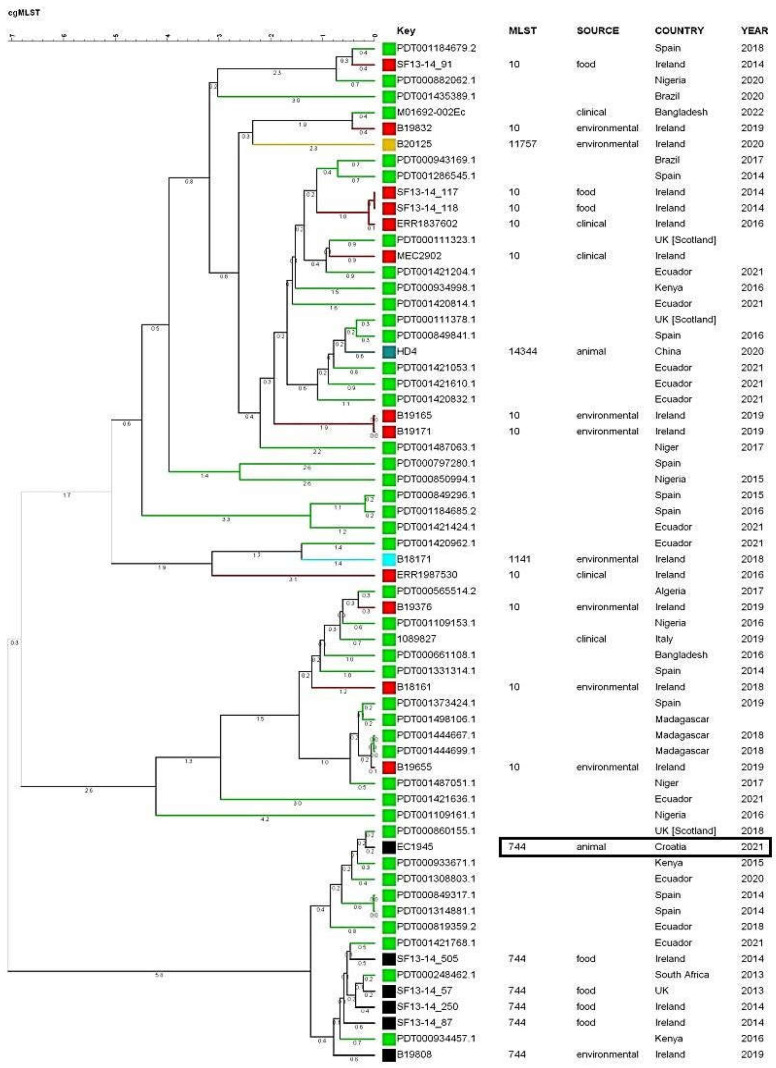
Dendrogram showing relationship of identified strain to the most related strains present in database used in this study calculated from cgMLST results. cgMLST profiles were generated using the 2513 core genes. Strains are also identified according to ST, source and country of origin species, and year of isolation. Complete linkage cluster analysis was used in the calculation with scaling factor of 100. Numbers indicated on the branches correspond to the number of allelic differences between strains multiplied by 100. Different colors indicate similarities between strains within same linkage cluster. Isolate from this study is in rectangle.

Figure 2 displays a dendrogram that illustrates the connection between the identified strain and the most closely related strains present in the database utilized in this study. This relationship includes information such as the source, country of origin, species, and year of isolation. The phylogenetic tree, constructed using allele numbers obtained from cgMLST analysis, is shown in Figure 3. The dendrogram classifies 60 patterns of strains, with numbers on the branches indicating the number of allelic differences multiplied by 100. The cgMLST analysis indicates that the *E. coli* strain ST744, which was isolated in Scotland, United Kingdom, is the closest strain to the EC1945 strain from this study, with only a 20 allele difference. Unfortunately, the isolate’s origin is unknown. The entire cluster of ST744 isolates, originating from food, environment, and animals, is within 120 allele differences.

## 4. Discussion

The overuse and misuse of antibiotics have resulted in the resistance of bacteria to all known antimicrobial classes, contributing to the emergence of multi-drug resistant (MDR) bacteria in both human medicine and veterinary practices [42,43,44]. Food-producing animals also play a significant role in the spread of antimicrobial resistance (AMR) as they act as a reservoir and intermediary for AMR between humans, animals, and the environment [45,46]. Additionally, food-producing animals serve as an important source of AMR genes, which have a direct impact on humans and the environment [47]. Colistin has been used in food-producing animals for decades, not only for the treatment of infections, but also as a growth promoter, so it is not surprising that widespread use of colistin has aided the spread of colistin resistance [10].

In recent years, there has been an increase in reports of plasmid-mediated colistin-resistant *E. coli* isolates in both livestock and humans. These isolates, in addition to being resistant to colistin, were found to be MDR [19,29]. More than 40 studies from 15 countries around the world have reported the presence of *E. coli* containing the *mcr-1* gene in pigs [19].

In this study, we analyzed the first mcr-1 positive, MDR *E. coli* strain isolated in pig caecal content in Croatia.

In 2021, MDR producing, O101:H10 *E. coli* isolate, belonging to ST744, that displayed a colistin MIC of 8 mg/L, was revealed. It was also resistant to gentamicin, ampicillin, cefepime, cefotaxime, ceftazidime, sulfamethoxazole, chloramphenicol, nalidixic acid, and ciprofloxacin (Table 1). Antimicrobial susceptibility testing confirmed ESBL phenotype. Resistance gene analysis provided clear evidence of the relationship between phenotype and genotype resistance.

The *mcr-1* gene was located on an IncX4-type plasmid, which is very common and part of an emerging plasmid expansion [32]. A total of 14 different plasmid incompatibility groups capable of carrying the *mcr-1* gene were identified. Over 90% of the worldwide identified plasmids belonged to IncX4, IncI2, and IncHI2 groups. The IncX4 group was the predominant Inc plasmid group carrying the *mcr-1* gene in Europe (48.9%), while the IncI2 group dominated in Asia (52.1%) [30]. The plasmid-mediated *mcr-1* gene confers colistin resistance by encoding a phosphoethanolamine transferase, which catalyzes the addition of a phosphoethanolamine moiety to the lipid-A lipopolysaccharide of the bacterium. A structural change in lipidA reduces its affinity for polymyxin [48].

Conjugative plasmids play a critical role in the dissemination of the *mcr-1* gene, and the IncI2 and IncX4 are the two leading plasmid types responsible for the global spread of colistin resistance [15,30]. Previous reports have shown that the IncX4 plasmid is highly transmissible, showing 10^2^–10^5^-fold higher transfer frequencies than IncFII plasmids [20]. It was also noted that IncX4 plasmids carrying the *mcr-1* gene are strikingly similar and show very high architectural conservation [49].

We identified typical conjugal modules on the EC1945_IncX plasmid, including auxiliary factor TaxA, relaxase TaxC, type IV secretion system genes (T4SS), pilX1-pilX11, and type IV coupling protein gene, taxB (T4CP) (Figure 1). Conjugative T4SS was found in other *mcr-1*-harbouring *E. coli* strains and could be responsible for horizontal gene transfer in conjugative plasmids [50]. Furthermore, a pair of 14-bp insert repeats were also found in the *ori*-T-like region, confirming that the EC1945_IncX4 plasmid is conjugative. 

Although the rapid transmission of IncX4 plasmids containing *mcr-1* is a major concern, the mechanisms that have enabled IncX4 plasmids to become successful vectors for the global spread of the *mcr-1* gene are mostly unclear. A recent study identified a novel transfer activator PixR, which is specific to IncX4 and IncX7 plasmids. *pixR* directly activates the expression of transfer genes, increases transfer capability, and results in the successful dissemination of IncX4 plasmids harboring the *mcr-1* gene [51].

Previous studies have shown that IncF plasmids have a conjugation transfer rate approximately 400 times lower than IncX4 plasmids, but a transfer rate 2.5 times higher than that of IncI plasmids [52]. Our study on *E. coli* EC1945 strain revealed the presence of four plasmids (Table 3). It has been noted that transfer rates can be influenced by the number of plasmids in the bacterial cell, with strains possessing two or more plasmids displaying faster transfer rates [53]. Therefore, we can confidently state that the plasmids detected in *E. coli* EC1945 are transferable.

The *mcr-1* gene was located on the IncX4 plasmid, while the *bla_CTX-M-1_* and *aadA5* genes were found on the IncI1 plasmid. Only *traT*, *sitA*, and *cea* virulence genes were found on plasmid contigs. However, the specific location of the other AMR and virulence genes is unclear as there is significant overlap on multiple plasmid contigs.

The *E. coli* EC1945 isolate was found to be resistant to cefotaxime, ceftazidime, and cefepime with MICs of 4 mg/L, 2 mg/L, and 8 mg/L, respectively. Resistance genes analysis confirmed that this *bla_CTX-M-1_* gene was found on the IncI1 plasmid. This gene belongs to CTX-M-type extended-spectrum β-lactamase, which confers resistance to extended-spectrum cephalosporins and is inhibited by clavulanic acid [54]. The *bla_CTX-M-1_* gene is the most frequently detected ESBL-type coded gene in animals in Europe, particularly in pigs [14,55,56,57].

We also detected the *bla_TEM-1b_* plasmid gene in *E. coli* EC1945 isolate, which is the most prevalent gene in the world associated with resistance to narrow-spectrum β-lactamases and inactivation of penicillin and aminopenicillins [58]. Previous studies from Australia, Denmark, Switzerland, the United States, and South Korea have described the *bla_TEM-1_* gene as the most common gene in clinical *E. coli* isolates responsible for β-lactam resistance in pigs [59,60,61,62,63].

Regarding fluoroquinolones resistance, we detected known double point mutations in gyrA (S83L, D87N) and parC (A56T, S80I). These have been closely associated with resistance to fluoroquinolones. This explains the MICs of 8 mg/L and >64 mg/L to ciprofloxacin and nalidixic acid, respectively. Previously, it was pointed out that fluoroquinolone resistance in the Enterobacteriaceae family has been mainly caused by point mutations in genes encoding DNA gyrase and topoisomerase IV (*gyrA, gyrB,* and *parC*) in quinolone resistance-determining regions (QRDRs) [64,65,66]. Mutations within *gyrA* and *parC* genes have been described in *E. coli* isolates originating from pigs and other food-producing animals [67,68,69].

In the *E. coli* EC1945 isolate, sulfamethoxazole resistance was mediated by the plasmid *sul2* gene. Resistance to sulphonamides, which are longstanding antimicrobials and the most commonly used class of antimicrobials in animal production, is prevalent globally [70]. Sulphonamide resistance in bacteria is mediated by *sul1, sul2,* and *sul3* genes, among which the *sul2* gene is most widely distributed in porcine, avian, or human *E. coli* sulphonamide-resistant isolates [61,63,71,72].

The *E. coli* EC1945 strain harbored the plasmid *catA1* gene, which was responsible for chloramphenicol resistance. Chloramphenicol is a broad-spectrum antibiotic that was extensively used in veterinary medicine in all major food-producing animals until concerns over its toxicity emerged [73]. Since 1994, it has been banned for use in food-producing animals in the European Union (EU) [74] and in many other countries, including the USA, Canada, Australia, Japan, and China. Phenicol resistance in *E. coli* of animal origin is mostly mediated by enzymatic inactivation of phenicols by chloramphenicol acetyltransferases encoded by *cat* genes [75]. The *catA1* gene was isolated worldwide in *E. coli* isolates from pigs [61,76,77,78].

Aminoglycosides are often used for treating complicated infections such as sepsis, pneumonia, meningitis, and urinary tract/abdominal infections, and are extremely important in both human and veterinary medicine [79]. Resistance to aminoglycosides in Gram-negative bacteria is mainly due to the production of aminoglycoside-modifying enzymes or modification of the ribosome by acquired 16S rRNA methyltransferases. Aminoglycoside-modifying enzymes catalyze the modification at −OH or −NH_2_ groups of the 2-deoxystreptamine nucleus or the sugar moieties and can be acetyltransferases (AACs), nucleotidyltranferases (ANTs), or phosphotransferases (APHs) [80]. Mutations in the genes responsible for the synthesis of these enzymes lead to the emergence of new enzyme variants and, consequently, resistance to a greater number of antibiotics of this class. Their ability to transfer at the molecular level as part of integrons, gene cassettes, transposons, or integrative conjugative elements results in the ability of this resistance mechanism to reach virtually all bacterial types [81]. It was reported that *E. coli* from pigs may be an important reservoir for the transfer of gentamicin resistance genes or bacteria to humans [82]. In our study, we identified two plasmid genes responsible for aminoglycoside resistance: *aac(3)-IId* and *aph (3*′)*-Ia*. AAC-3 enzymes catalyze the acetylation of the –NH2 group in an aminoglycoside antibiotic at the third position [80] and are responsible for resistance to apramycin, gentamicin, netilmicin, tobramycin, sisomicin, and dibekacin [83]. APHs catalyze the transfer of a phosphate group to the aminoglycoside molecule [84]. The APH(3′)-I subclass shows a resistance profile including kanamycin, neomycin, paromomycin, ribostamycin, and lividomycin, and is composed of three enzymes that are widely distributed mainly among gram-negatives [85].

The Plasmid *sitABCD* gene was also found in *E. coli* EC1945 isolate. The SitABCD system mediates the transport of iron and manganese. Its ability to obtain manganese contributes to the resistance to oxidative stress and protection against agents such as hydrogen peroxide [86].

The *E. coli* strain EC1945 screened in this study belonged to ST744. It is a single-locus variant of ST10, belonging to clonal complex (CC) 10, and A phylogenetic *E. coli* group. This clonal complex is widely disseminated [87] and ranks as the third most common extraintestinal pathogenic *E. coli* (ExPEC) in a systematic review of human studies [88]. ExPEC strains are associated with a variety of infections, including urinary tract infections (UTI), neonatal meningitis, septicemia, diverse intraabdominal infection, pneumonia, osteomyelitis, and soft-tissue infection [89]. It has been previously emphasized that the existence and high prevalence of MDR *E. coli* isolates harboring the *mcr-1* gene in the ST10-related population is due to their intrinsic ability to acquire AMR genes. This population of commensal *E. coli* isolates then plays a crucial role as a reservoir for these genes [30]. MDR *E. coli* ST744 isolates have been found in multiple animal species, environments, and diseased humans worldwide. ST744, *mcr-1* positive, isolates have been obtained from human bloodstream infection in Denmark [90], human fecal samples in China and Brazil [91,92], UTI in Portugal and Brazil [93,94], and sputum/body fluid in China [95]. *E. coli* ST744 strains have also been reported in *mcr-1-*positive isolates from poultry in Romania and Lebanon [96,97], weaning pigs in Japan [98], while mcr-3-producing *E. coli* ST744 isolate was reported in veal calves in France [99]. Additionally, previous studies have emphasized that *E. coli* ST744 isolates consistently had mutations in QRDRs of *gyrA* and *parC* genes [100], as demonstrated in the isolate in our study.

According to the cgMLST analysis, the E. coli strain ST744 found in Scotland is very similar to the EC1945 strain from our study, with only a difference of 20 alleles. These 2 strains form a separate cluster and are 40 alleles different from the main linkage cluster that consists of 2 strains from Ecuador and Spain, and one strain from Kenya (Figure 3). The entire cluster of ST744 isolates is within 120 allele differences, indicating quite a similarity of ST744 strains.

We analyzed the virulence factor-encoding genes (VFGs) present in the sequenced *E. coli* EC1945 isolate to determine its potential to cause disease. The virulence factors (VFs) present in bacteria, such as adhesins, toxins, siderophores, capsules, hemolysins, and invasins, largely determine the virulent potential of bacteria. These VFs help the microorganism to avoid host defenses, invade, colonize, and cause disease [101]. According to the virulence profile (Table 2), *E. coli* EC1945 had extra-intestinal pathogenic potential, primarily due to the presence of *fimH, gad, hlyE, hlyF, iss,* and *TraT* genes. Previously, it was described that FimH adhesion located at the tip of the bacterial type 1 fimbrium mediates the binding to urothelial cells and prevents bacterial washout by micturition [102,103]. The *gad* gene encodes the enzyme glutamate decarboxylase and is responsible for *E. coli* survival of low pH exposure and passage through the stomach after ingestion [104]. Hemolysin E is one of the numerous virulence factors of *E. coli* strains responsible for ExPEC infections. It is a pore-forming toxin that lyses mammalian erythrocytes. It is also toxic toward cultured mammalian cells and induces apoptosis in macrophages [105,106]. Hemolysin F was supposed to be an avian hemolysin associated with avian pathogenic *E. coli* [107], and it has been shown that plays an important role in the virulence of ExPEC [108]. Another very important ExPEC virulence gene is *iss*, which is responsible for surviving the bactericidal effects of the complement system [109,110]. It is associated with the avian pathogenic *E. coli* (APEC) subpathotype of ExPEC and is found to occur in around 60% of uropathogenic and neonatal meningitis-associated *E. coli* strains [111,112]. The *TraT* is another gene that was described as significant VF in neonatal meningitis *E. coli* (NMEC), sepsis-associated *E. coli* (SEPEC), uropathogenic *E. coli* (UPEC), and APEC in the ExPEC group. It expresses a transfer protein that inhibits the classical pathway of complement activation [113].

*E. coli* strains belonging to the O101:H10 serotype and CC10 were previously isolated from human bloodstream in Spain (ESBL phenotype) [114], UTI in Taiwan (carbapenemase phenotype) [115], and Argentina (ESBL phenotype) [116].

## 5. Conclusions

We characterized the first mcr-1 positive *E. coli* strain isolated in Croatia. The present study provides a detailed analysis of the MDR *E. coli* EC1945 strain harboring the *mcr-1* gene isolated from pig caecal content. The *mcr-1* gene was located on the IncX4 plasmid, which is the predominant Inc plasmid group carrying the *mcr-1* gene in Europe. Apart from IncX4, the *E. coli* EC1945 strain also harbored three other transferable Inc plasmid groups, namely IncI1, IncFIB, and IncFII. However, due to overlapping plasmid contigs, we could not reconstruct them in this study. Our data indicate that the *E. coli* EC1945 isolate exhibits phenotypic and genotypic resistance to nine antimicrobial classes, and possesses 14 virulence genes, which classify it as MDR and ExPEC isolate. Regarding the Achtman scheme, our strain was classified as ST744, which is a single locus variant of ST10, commensal *E. coli* isolates then play a crucial role as a gene reservoir. Further efforts are needed to identify mcr-1-carrying *E. coli* isolates in Croatia, especially in food-producing animals to identify such gene reservoirs, as we think that this strain is not an isolated case.

## Figures and Tables

**Table 1:** List of class, antimicrobials, MICs, ECOFFs, and resistant genes for *E. coli* EC1945 isolate.

Class	Antibimicrobial	MIC (mg/L)	ECOFF (mg/L)	R/S	Gene Detected (%)
Macrolides	AZI	4	16 *	S	-
Aminoglycosides	AMI	≤4	8	S	-
	GEN	>16	2	R	*aac(3)-IId* (99.88) *aph (3′)-Ia* (100)
Glycylcyclines	TGC	≤0.25	0.5	S	-
Aminopenicillins	AMP	>32	8	R	*bla_TEM-1B_* (100) *bla_CTX-M-1_*(100)
Cephalosporins 2nd generation	FOX	4	8	S	-
Cephalosporins 4th generation	FEP	8	0.25	R	*bla_CTX-M-1_* (100)
Cephalosporins 3rd generation	TAZ	2	0.5	R	*bla_CTX-M-1_* (100)
	FOT	64	0.25	R	*bla_CTX-M-1_* (100)
Cephalosporins 3rd generation/β-lactamase inhibitor	T/C	≤0.12	0.5	S	-
	F/C	≤0.06	0.25	S	-
Carboxypenicillins	TRM	8	16	S	-
Carbapenems	ETP	0.03	0.03 *	S	-
	MERO	≤0.03	0.125	S	-
	IMI	≤0.12	0.5	S	-
Polymyxin	COL	8	2	R	*mcr-1.1* (100)
Tetracyclines	TET	≤2	8	S	-
Antifolates	TMP	≤0.25	2	S	-
Sulfonamides	SMX	>512	64	R	*sul2* (100)
Phenicols	CHL	>64	16	R	*catA1* (99.85)
Quinolone	NAL	>64	8	R	*gyrA*(p.S83L) *gyrA*(p.D87N) *parC*(p.A56T) *parC*(p.S80I)
Fluoroquinolones	CIP	8	0.06	R	

* Tentative ECOFF. AZI—azithromycin, AMI- amikacin, GEN- gentamicin, TGC-tigecycline, AMP- ampicillin, FOX- cefoxitin, FEP- cefepime, TAZ- ceftazidime, T/C- ceftazidime/clavulanic acid, FOT- cefotaxime, F/C- cefotaxime/clavulanic acid, TRM- temocillin, ETP- ertapenem, MERO- meropenem, IMI- imipenem, COL-colistin, TET-tetracycline, TMP-trimethoprim, SMX- sulfamethoxazole, CHL- chloramphenicol, NAL- nalidixic acid, CIP- ciprofloxacin, MIC—minimal inhibitory concentration.

**Table 2:** Virulence genes detected in *E. coli* EC1945 isolate.

Virulence Factor	Identity (%)	Protein Function
cea	100	Colicin E1
cia	99.32	Colicin Ia
cib	100	Colicin Ib
cma	100	Colicin M
csgA	100	Curlin major subunit CsgA
fimH	100	Type 1 fimbriae
gad	100	Glutamate decarboxilase
hlyE	100	Hemolysin E
hlyF	99.91	Hemolysin F
iroN	99.95	Enterobactin siderophore receptor protein
iss	100	Increased serum survival
sitA	100	Iron transport protein
terC	99.9	Tellurium ion resistance protein
traT	98.71	Outer membrane protein complement resistance

**Table 3:** PlasmidFinder, ResFinder, and VirulenceFinder results on plasmid contigs.

Contig Number	RFPlasmid Prediction (%)	PlasmidFinder (%)	ResFinder (%)	VirulenceFinder (%)
40	97	IncFII (96.56)	-	*traT* (100)
51	89	-	-	-
61	90	IncI1 (99.3)	*bla_CTX-M-1_* (100); *aadA5* (100)	-
68	87	-	-	-
79	84	IncX4 (99.97)	*mcr-1.1* (100)	-
84	88	-	-	-
87	95	-	*bla_TEM-1B_* (100); *aac (3)-IId* (99.88)	-
100	80	-	*sitABCD* (99.62)	*sitA* (100)
105	97	-	-	*cea* (100)
106	80	-	*sul2* (100)	-
107	98	-	-	-
110	96	-	*catA1* (99.85)	-
112	80	IncFIB (98.39)	-	-
114	97	-	-	-
157	86	/	*aph (3’)-Ia* (100)	/
173	100	/	/	/

**Table 4:** MLST analysis of *E. coli* EC1945 isolate/Achtman scheme.

Sample	MLST Loci (Allele Length/Number of Repeats)														ST
	*adk*		*fumC*		*gyrB*		*icd*		*mdh*		*purA*		*recA*		744
*E. coli* EC1945	536	10	469	11	460	135	518	8	452	8	478	8	510	2	
